# High visceral adiposity and low skeletal muscle mass independently predict the development of acute cholecystitis in patients with gallstones: a retrospective cohort study

**DOI:** 10.3389/fmed.2025.1724416

**Published:** 2025-12-03

**Authors:** Abdullah Enes Ataş, Şeyma Ünüvar

**Affiliations:** 1 Department of Radiology, Faculty of Medicine, Necmettin Erbakan University, Konya, Türkiye; 2 Department of Radiology, Konya City Hospital, Konya, Türkiye

**Keywords:** acute cholecystitis, visceral adiposity, sarcopenia, body composition, cholelithiasis

## Abstract

**Background:**

While cholelithiasis is common, predicting which patients will develop acute cholecystitis (AC) remains a clinical challenge, as traditional risk factors like Body Mass Index (BMI) lack specificity. This study aimed to determine if CT-derived visceral adiposity and sarcopenia can predict the transition from asymptomatic cholelithiasis to clinical AC.

**Methods:**

This retrospective cohort study included 622 adult patients with cholelithiasis, identified via CT scans performed between 2015 and 2025. Patients were divided into an AC group (*n* = 164), who developed AC during follow-up, and a control group (*n* = 458) who did not. Body composition metrics, including visceral fat area (VFA) and skeletal muscle index (SMI), were quantified from axial CT images at the L3 vertebral level. A multivariate logistic regression analysis was performed to identify independent predictors of developing AC.

**Results:**

The AC group had significantly higher VFA and lower SMI compared to the control group. Multivariate analysis identified several independent predictors for developing AC, including hypertension (OR: 2.71, *p* < 0.001), hyperlipidemia (OR: 2.88, *p* < 0.001), and a 10 cm^2^ increase in VFA (OR: 1.34, *p* < 0.001). In contrast, a per-unit increase in SMI was found to be protective (OR: 0.94, *p* = 0.001). The predictive model demonstrated strong discriminatory power with an Area Under the Curve (AUC) of 0.857.

**Conclusion:**

Visceral adiposity and sarcopenia are strong, independent predictors of the development of AC. These findings suggest that AC is a complication of a systemic metabolic state and that CT-based body composition analysis is a valuable tool for risk stratification in patients with cholelithiasis.

## 1 Introduction

Cholelithiasis is a highly prevalent condition worldwide, affecting approximately 10–20% of the population. The most common complication is acute cholecystitis (AC), which occurs in 1–3% of symptomatic patients annually and constitutes 3–10% of all hospital admissions for acute abdominal pain (1, 2).

Although the traditional risk factors for gallstone formation—the “4Fs” (Female, Fat, Forty, Fertile) and body mass index (BMI)—are well-established, they lack the specificity to predict which patients with cholelithiasis will progress to AC (3). BMI is an particularly crude metric as because it fails to differentiate between metabolically distinct fat depots, such as subcutaneous and visceral fat, and does not account for muscle mass. This limitation highlights the need for more granular biomarkers to predict disease progression (4).

Growing body of evidence indicates that central or visceral adiposity, rather than general obesity, is a more potent driver of metabolic diseases (5–7). Visceral adipose tissue (VAT) is not an inert storage depot but a metabolically active organ that releases a cascade of pro-inflammatory cytokines (e.g., TNF-α, IL-6) and free fatty acids directly into the portal circulation (8–10). This process creates a state of chronic, low-grade systemic inflammation and hepatic insulin resistance, which in turn promotes the secretion of lithogenic, cholesterol-supersaturated bile (11). Accordingly, studies have strongly associated higher VAT and an elevated VAT-to-Subcutaneous Adipose Tissue (SAT) ratio with the risk of cholelithiasis itself (12).

Furthermore, sarcopenia, the age- or disease-related loss of muscle mass and function, is increasingly recognized as a robust predictor of adverse outcomes across numerous medical and surgical disciplines (13–16). The measurement of muscle mass (Skeletal Muscle Index, SMI) and quality (Skeletal Muscle Radiodensity, an indicator of myosteatosis) via CT scans at the L3 vertebral level is a validated method for opportunistic sarcopenia assessment (17). Sarcopenia has also been strongly linked to poor outcomes in patients with established AC, including intensive care unit admission, longer hospital stays, increased mortality in sepsis, and higher rates of recurrence following conservative management (18).

While it is established that VAT is linked to stone formation and sarcopenia to adverse outcomes following AC, a critical knowledge gap exists. It remains unknown whether these body composition phenotypes can predict the initial inflammatory event that transitions asymptomatic cholelithiasis into clinical AC. Our hypothesis is that the pro-inflammatory state induced by high VAT lowers the threshold for an obstructive stone to trigger an acute inflammatory cascade within the gallbladder wall.

We purpose this pathophysiological mechanism operates as follows: systemic inflammatory mediators released from VAT “prime” the gallbladder, causing it to mount an exaggerated inflammatory response to a mechanical trigger from an impacted stone. Furthermore, we postulate that the impaired physiological reserve associated with sarcopenia may also contribute to this risk. This study is designed to test these hypotheses by examining body composition prior to the onset of AC.

## 2 Materials and methods

### 2.1 Study design and ethics statement

This retrospective study was approved by the Necmettin Erbakan University Ethics Committee (Decision no. 2025/5950). Written informed consent requirements were waived. Patient data obtained from the hospital information system and radiology archive were anonymized and included in the study. This study was conducted in accordance with the Declaration of Helsinki and Good Clinical Practices.

### 2.2 Patient population

A retrospective cohort study was conducted utilizing a dataset derived from the clinical data and radiological images of patients who presented to our tertiary university hospital between January 1, 2015, and January 1, 2025.

- Inclusion criteria: (1) Adult patients (age = 18 years); (2) Patients with incidentally detected gallstones documented on abdominal CT scan performed for any clinical indication (without acute cholecystitis or acute biliary pathology); (3) Availability of at least 12 months of follow-up data after the index CT scan. Minimum follow-up of 12 months was required, as this period is generally considered clinically sufficient to observe the potential progression from asymptomatic cholelithiasis to symptomatic disease or complications such as AC, while balancing data availability in a retrospective cohort.
- Exclusion criteria: (1) Prior history of cholecystectomy or AC; (2) Acalculous cholecystitis; (3) Confounding causes of gallbladder wall thickening (e.g., heart failure, liver failure, hypoalbuminemia); (4) CT images with significant artifacts that would preclude accurate body composition analysis.
- Cohort definition: Patients were followed retrospectively from the date of their index CT scan.
- AC group (Cases): Patients hospitalized with a primary diagnosis of AC, confirmed by clinical (e.g., Murphy’s sign, fever), laboratory (e.g., leukocytosis), and imaging (e.g., gallbladder wall thickening, pericholecystic fluid) findings consistent with the Tokyo Guidelines.
- Control group (non-AC): Patients from the same cohort who did not develop AC during the follow-up period. To ensure an adequate observation period for the development of the primary outcome, a minimum follow-up of 12 months was required. The median follow-up duration for the entire cohort was 76 months.

Data were collected from patients regarding age, sex, and the presence of comorbidities, including Diabetes Mellitus, Hypertension, and Hyperlipidemia/Dyslipidemia. Additionally, Body Mass Index (BMI, kg/m^2^) was calculated using the height (m) and weight (kg) recorded at the time of the index CT scan.

### 2.3 CT imaging and body composition analysis

CT imaging was performed on patients using either a 256-slice Somatom Drive or a 64-slice Somatom Definition scanner (Siemens AG, Erlangen, Germany). The analysis was conducted on a single axial slice from non-contrast or contrast-enhanced abdominal CT scans with a slice thickness of 1.5 mm. This slice was taken at the midpoint of the L3 vertebral body, a validated level for comprehensive body composition assessment (19).

**FIGURE 1:**
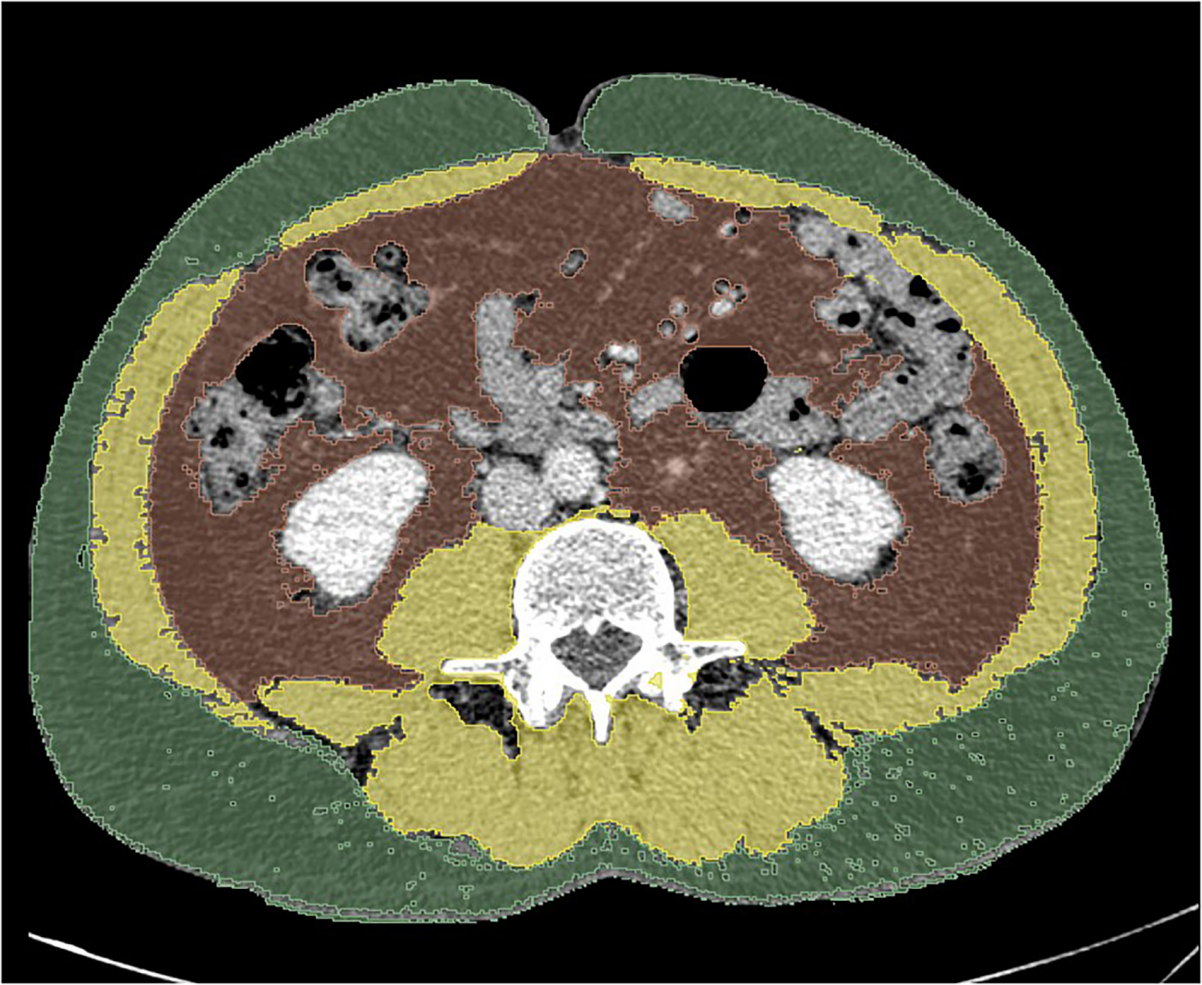
Measurement of body composition parameters on an axial CT slice at the L3 level. Subcutaneous fat area (green), skeletal muscle area (yellow), and visceral fat area (brown).

The open-source freeware 3D Slicer (20), was used as the segmentation tool to measure tissue areas (Figure 1). The following Hounsfield Unit (HU) thresholds were established:

- Adipose Tissue (VAT and SAT): –190 to -30 HU
- Skeletal Muscle (SMA and Psoas): –29 to + 150 HU

Measurements (in cm^2^):

- Visceral Fat Area (VFA): The area of intra-abdominal fat.
- Subcutaneous Fat Area (SFA): The area of fat superficial to the muscular wall.
- Total Skeletal Muscle Area (SMA): The cross-sectional area of all skeletal muscles at the L3 level (psoas, erector spinae, quadratus lumborum, obliques, rectus abdominis).
- Psoas Muscle Area (PMA): The combined area of the right and left psoas muscles.
- Abdominal Circumference (AC): Measured at the L3 level.

Muscle quality measurement:

- Skeletal Muscle Radiodensity (SMRD): The mean HU value of the entire SMA, serving as an indicator of myosteatosis (intramuscular fat infiltration).

Derived body composition indices:

- Skeletal Muscle Index (SMI): SMA (cm^2^)/height (m^2^).
- Psoas Muscle Index (PMI): PMA (cm^2^)/height (m^2^); an alternative marker for sarcopenia.
- Visceral-to-Subcutaneous Adipose Tissue Ratio (VSR): A key indicator of central adiposity distribution.
- Visceral Adipose Tissue-to-Skeletal Muscle Ratio (VMR): An index of sarcopenic obesity.

### 2.4 Statistical analysis

Descriptive statistics were presented as mean ± standard deviation (SD) for normally distributed continuous variables and as median (interquartile range, IQR) for non-normally distributed variables. Categorical variables were summarized as frequencies and percentages (n,%). The normality of data distribution was assessed using the Shapiro-Wilk test and visual inspection of histograms. It was determined that height, VFA, PMA, PMI, VSR, and VMR were not normally distributed, whereas all other parameters followed a normal distribution.

For intergroup comparisons, the Student’s *t*-test was applied to normally distributed continuous data, while the Mann-Whitney U test was used for non-normally distributed data. The Chi-square test or Fisher’s exact test was employed for the comparison of categorical variables.

To identify independent predictors of AC, a binary logistic regression model was constructed. Variables with a *p* < 0.10 in the univariate analysis, along with known confounders (age, sex, BMI), were included in the model. The results were reported as Odds Ratios (OR) with their 95% Confidence Intervals (CI). To ensure the stability of the logistic regression model, multicollinearity among the independent variables was assessed using the Variance Inflation Factor (VIF). A VIF value exceeding 5 was considered indicative of significant multicollinearity. A *p* of < 0.05 was considered statistically significant. All statistical analyses were performed using the open-source software Jamovi v2.6 (Sydney, Australia).

## 3 Results

### 3.1 Descriptive statistics of the study population

The study cohort comprised a total of 622 patients with cholelithiasis, who were divided into two groups: the AC group (*n* = 164) and the control group (*n* = 458). The demographic, clinical, and body composition characteristics of both groups were analyzed (Tables 1, 2).

**TABLE 1:** Demographic characteristics and comorbidities of the ac and control groups.

	AC group (*n* = 164)	Control group (*n* = 458)	*p*-value
Age (years, mean ± SD)	63.24 ± 12.01	59.31 ± 15.11	< 0.001
Gender [female, *n* (%)]	88 (53.7)	223 (48.7)	0.354
BMI (kg/m^2^, mean ± SD)	29.1 ± 5.2	28.3 ± 4.9	0.017
Diabetes mellitus [*n* (%)]	60 (36.5)	110 (24.0)	0.002
Hypertension [*n* (%)]	108 (65.9)	203 (44.3)	< 0.001
Hyperlipidemia [*n* (%)]	97 (59.1)	176 (38.4)	< 0.001

**TABLE 2:** Descriptive statistics from the patient cohort.

	Mean	SD	Median	IQR
Age	60.35	14.3256	60.000	19.750
Height (m)	1.68	0.0958	1.680	0.150
Weight (kg)	81.26	17.1842	79.950	23.350
Body mass index (kg/m^2^)	28.61	5.0048	28.500	6.475
Visceral fat area (cm^2^)	142.62	58.0486	137.550	75.925
Subcutaneous fat area (cm^2^)	160.57	59.1841	158.100	84.025
Skeletal muscle area (cm^2^)	143.07	34.1815	140.250	45.750
Psoas muscle area (cm^2^)	14.44	4.5664	13.900	6.200
Abdominal waist (cm)	94.54	12.1654	94.400	16.625
Skeletal muscle radiodensity (HU)	32.94	9.2425	32.900	12.800
Skeletal muscle index (cm^2^/m^2^)	50.43	10.7728	49.970	15.008
Psoas muscle index (cm^2^/m^2^)	5.09	1.5153	4.975	2.115
Visceral/subcutaneous fat ratio	1.06	0.7415	0.859	0.718
Visceral fat/skeletal muscle ratio	1.08	0.5874	0.983	0.685

The mean age of the AC group was significantly higher than that of the control group (63.24 ± 12.01 years vs. 59.31 ± 15.11 years, respectively) (*p* < 0.001). The AC group consisted of 76 (46.3) males and 88 (53.7%) females, while the control group included 235 (51.3) males and 223 (48.7%) females. Patients in the AC group also demonstrated a higher mean BMI compared to the control group (29.1 ± 5.2 kg/m^2^ vs. 28.3 ± 4.9 kg/m^2^) (*p* = 0.017).

The prevalence of comorbidities was notably higher in the AC group. Diabetes mellitus was present in 36.5% (*n* = 60) of AC patients compared to 24% (*n* = 110) in the control group (*p* = 0.002). Similarly, hypertension was observed in 65.9% (*n* = 108) of the AC group vs.44.3% (*n* = 203) of controls, and hyperlipidemia was present in 59.1% (*n* = 97) of AC patients vs.38.4% (*n* = 176) of controls (*p* < 0.001).

### 3.2 Comparison of CT-derived body composition parameters

The AC group exhibited a significantly higher VFA (192.352 ± 61.5073 cm^2^) compared to the control group (124.819 ± 44.9932 cm^2^) (*p* < 0.001). Conversely, there was no statistically significant difference in the SFA between the AC group (156.760 ± 59.4039 cm^2^) and the control group (161.936 ± 59.1105 cm^2^) (*p* = 0.337). The abdominal waist circumference was also significantly greater in the AC group (100.563 ± 12.6750 cm) than in the control group (92.389 ± 11.2315 cm) (*p* < 0.001).

Regarding muscle composition, the AC group had a significantly lower SMA (135.033 ± 33.3310 cm^2^ vs. 145.943 ± 34.0582 cm^2^, *p* < 0.001), PMA (13.704 ± 4.2087 cm^2^ vs. 14.701 ± 4.6641 cm^2^, *p* = 0.045), SMRD (29.966 ± 9.2166 HU vs. 34.005 ± 9.0261 HU, *p* < 0.001), SMI (47.136 ± 10.4902 cm^2^/m^2^ vs. 51.614 ± 10.6373 cm^2^/m^2^, *p* < 0.001), and PMI (4.798 ± 1.4384 cm^2^/m^2^ vs. 5.200 ± 1.5295 cm^2^/m^2^, *p* = 0.006) when compared to the control group.

Furthermore, the ratios calculated from these parameters were significantly higher in the AC group. The VSR was 1.419 ± 0.7218 in the AC group vs.0.938 ± 0.7072 in the control group (*p* < 0.001), and the VMR was 1.542 ± 0.7148 in the AC group compared to 0.916 ± 0.4279 in the control group (*p* < 0.001). Statistical significance was determined using the *t*-test or the Mann-Whitney U test as appropriate (Table 3).

**TABLE 3:** Comparison of CT-derived body composition parameters between patients with ac and control groups.

Parameter	AC group (*n* = 164)	Control group (*n* = 458)	*p*-value
Visceral fat area (cm^2^)	192.352 ± 61.5073	124.819 ± 44.9932	< 0.001*
Subcutaneous fat area (cm^2^)	156.760 ± 59.4039	161.936 ± 59.1105	0.337*
Skeletal muscle area (cm^2^)	135.033 ± 33.3310	145.943 ± 34.0582	< 0.001*
Psoas muscle area (cm^2^)	13.704 ± 4.2087	14.701 ± 4.6641	0.045**
Abdominal waist (cm)	100.563 ± 12.6750	92.389 ± 11.2315	< 0.001*
Skeletal muscle radiodensity (HU)	29.966 ± 9.2166	34.005 ± 9.0261	< 0.001*
Skeletal muscle index (cm^2^/m^2^)	47.136 ± 10.4902	51.614 ± 10.6373	< 0.001*
Psoas muscle index (cm^2^/m^2^)	4.798 ± 1.4384	5.200 ± 1.5295	0.006**
Visceral/subcutaneous fat ratio	1.419 ± 0.7218	0.938 ± 0.7072	< 0.001**
Visceral fat/skeletal muscle ratio	1.542 ± 0.7148	0.916 ± 0.4279	< 0.001**

*t-test, **Mann-Whitney U test.

### 3.3 Logistic regression analysis of factors associated with ac

In the univariate logistic regression analysis (Table 4), a 10-year increase in age, a per-unit increase in BMI, the presence of diabetes mellitus, hypertension, and hyperlipidemia were all significantly associated with AC (*p* = 0.002, *p* < 0.001, *p* < 0.001, respectively). Additionally, several body composition parameters were significant predictors, including VFA, SMRD, SMI, and PMI (*p* < 0.001, *p* < 0.001, *p* < 0.001, *p* = 0.004, respectively). Gender was not found to have a significant association (*p* = 0.312).

**TABLE 4:** Multivariate logistic regression analysis of factors associated with acute cholecystitis.

Variable	Univariate logistic regression		Multivariate logistic regression (*R*^2^ = 0.333)	
	OR [95% (CI)]	*p*-value	OR [95% (CI)]	*p*-value
Age (per 10-year increase)	1.21 [0.06–1.37]	**0.003**	0.81 [0.66–0.99]	0.048
Gender (female)	1.15 [0.88–1.50]	0.312		
BMI (per unit increase)	1.04 [1.00–1.08]	**0.017**	0.94 [0.90–0.99]	0.033
Diabetes mellitus	1.82 [1.24–2.67]	**0.002**	1.56 [0.94–2.58]	0.081
Hypertension	2.42 [1.67–3.51]	**< 0.001**	2.71 [1.70–4.31]	< 0.001
Hyperlipidemia	2.32 [1.61–3.33]	**< 0.001**	2.88 [1.79–4.63]	< 0.001
VFA (per 10 cm^2^ increase)	1.29 [1.23–1.36]	**< 0.001**	1.34 [1.26–1.42]	< 0.001
SMRD (per unit increase)	0.95 [0.93–0.97]	**< 0.001**	1.00 [0.97–1.03]	0.728
SMI (per unit increase)	0.96 [0.94–0.97]	**< 0.001**	0.94 [0.91–0.97]	0.001
PMI (per unit increase)	0.83 [0.73–0.94]	**0.004**	1.17 [0.94–1.45]	0.157

Bold values indicate variables included in the multivariate analysis (*p* < 0.05). BMI, Body Mass Index; CI, Confidence Interval; OR, Odds Ratio; PMI, Psoas Muscle Index; SMI, Skeletal Muscle Index; SMRD, Skeletal Muscle Radiodensity.

An assessment of multicollinearity was conducted before the multivariate analysis. All predictor variables demonstrated a Variance Inflation Factor (VIF) between 1.0 and 2.5, confirming the absence of significant collinearity. To identify independent predictors, a multivariate logistic regression analysis was performed (*R*^2^ = 0.333). The results indicated that hypertension (OR: 2.71, 95% CI: 1.70 – 4.31, *p* < 0.001) and hyperlipidemia (OR: 2.88, 95% CI: 1.79–4.63, *p* < 0.001) were strong independent risk factors for AC. Among the body composition metrics, a 10 cm^2^ increase in VFA was associated with increased odds (OR: 1.34, 95% CI: 1.26–1.42, *p*– < 0.001), while a per-unit increase in SMI was associated with decreased odds (OR: 0.94, 95% CI: 0.91–0.97, *p* = 0.001) of having AC. A 10-year increase in age (OR: 0.81, 95% CI: 0.66–0.99, *p* = 0.048) and a per-unit increase in BMI (OR: 0.94, 95% CI: 0.90 – 0.99, *p* = 0.033) were also found to be independent protective factors. In the multivariate model, gender, diabetes mellitus, SMRD, and PMI were not statistically significant predictors.

**FIGURE 2:**
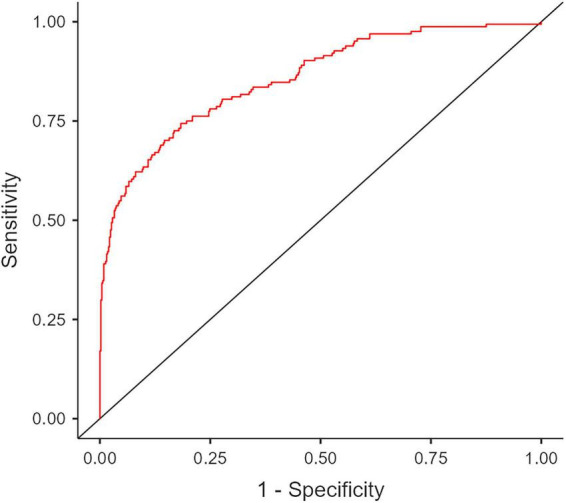
Receiver Operating Characteristic (ROC) curve analysis of the final multivariate model for predicting acute cholecystitis in patients with gallstones. The calculated Area Under Curve (AUC) is 0.857.

The predictive performance of the final multivariate model was evaluated using a Receiver Operating Characteristic (ROC) curve. The model demonstrated strong predictive ability, with an Area Under the Curve (AUC) of 0.857 (Figure 2).

## 4 Discussion

### 4.1 Visceral Adiposity as an independent pro-inflammatory risk factor for acute cholecystitis

The most salient finding of this study is that VFA stands as a robust risk factor for developing AC, independent of general obesity as measured by BMI. This strongly supports the initial hypothesis that pro-inflammatory cytokines released from VAT predispose the gallbladder to inflammation. The results, therefore, suggest that the development of AC is not simply a consequence of mechanical obstruction, but is instead initiated against a pre-existing metabolic and inflammatory background.

### 4.2 The prognostic significance of body composition in gallbladder disease

It is well-established that obesity and body composition are risk factors for cholelithiasis, particularly through their association with metabolic syndrome (21). This effect is largely attributed to VAT rather than SAT, as VAT is considered more metabolically active (22). It has been shown that a one standard deviation increase in VFA corresponds to a 4.26-fold increase in the risk of developing cholelithiasis (23). Indeed, studies have shown that an increased VFA not only elevates the risk of gallstones but also prolongs the length of hospital stay for gallstone-related admissions (24, 25).

For instance, Kim et al. (18) demonstrated that in patients with AC, sarcopenia prolongs the length of hospital stay and increases the likelihood of requiring intensive care. Furthermore, a low PMI was identified as a risk factor for the recurrence of AC (26). In addition, in AC, low muscle mass and high visceral fat are associated not only with a longer length of hospital stay but also with decreased 1-year survival (24).

Shifting the focus from these prognostic markers to etiology our study found that, while the comparison between groups showed lower SMA and SMI in the AC cohort, the multivariate analysis identified high SMI as an independent protective factor against the development of AC. This is a significant finding, suggesting that sarcopenia is not only associated with poor outcomes after the onset of AC but may also influence the risk of developing the disease itself. It is conceivable that having a good muscle reserve may provide a form of physiological protection against the inflammatory cascade triggered by visceral fat.

While the detrimental impact of sarcopenia on outcomes in patients with established acute cholecystitis is well-documented (18, 25, 26), its role prior to the onset of the disease has remained unexplored. A novel contribution of our study is the demonstration that sarcopenia, as indicated by a lower SMI, is not merely a marker of poor prognosis following an inflammatory event, but is also an independent risk factor for the initial development of AC. This finding reframes sarcopenia from being solely a prognostic indicator of frailty to being a potential contributing factor in the pathophysiology of the disease itself. Our findings suggest that a robust skeletal muscle reserve may confer a form of systemic physiological resilience, potentially counteracting the chronic, low-grade inflammatory state propagated by visceral adipose tissue and thereby raising the threshold for an obstructive stone to trigger a severe inflammatory cascade in the gallbladder wall.

### 4.3 Visceral obesity as a gender-independent risk factor

It is well-established that gallstones and AC are more prevalent in women, with female sex long considered an independent risk factor for cholelithiasis (26, 27). Anthropometric indicators such as waist-to-height ratio, waist-to-hip ratio, VAT, and BMI have been associated with gallstone risk in women, with waist-to-height ratio in particular suggested to be a better predictor (22). However, large cohort studies have demonstrated that an increase in VAT is strongly associated with gallstone risk, independent of sex (12, 28). This suggests that visceral adiposity could be considered a predictive risk factor for both gallstones and AC. Similarly, our study found that gender was not a significant risk factor for the development of AC. This suggests that metabolic phenotypes, such as visceral obesity and sarcopenia, which can be present in both sexes, play a more fundamental role in determining risk than gender itself.

In our multivariate model, increased age and BMI surprisingly emerged as independent protective factors, a finding confirmed to be statistically robust after VIF analysis ruled out significant multicollinearity. This paradox likely reflects the inadequacy of BMI as a risk metric once visceral fat is controlled for, alongside potential confounding from the anti-inflammatory effects of medications commonly used in older populations. Therefore, these findings do not challenge our primary conclusion but instead highlight a complex metabolic interplay, reinforcing that body composition is a more critical risk determinant than traditional anthropometric measures.

### 4.4 Acute cholecystitis as a manifestation of metabolic syndrome

Diabetes mellitus is a recognized risk factor for cholelithiasis. Concurrently, insulin resistance is believed to promote gallstone formation via mechanisms like compromised gallbladder function and reduced bile acid synthesis (22, 29). Chronic conditions are typically more prevalent in obese populations, and a study has reported a higher incidence of hypertension among patients with gallstones. Regarding hyperlipidemia, the rise in lipid accumulation products is generally reflected as an increase in visceral fat (30, 31). Correspondingly, Wu et al. found in the same study that the risk of gallstones increased by a factor of 2.48 with rising levels of lipid accumulation product (31).

The finding in our study that hypertension and hyperlipidemia are potent, independent risk factors for AC, alongside VFA, highlights the close connection between the pathophysiology of the disease and metabolic syndrome. This supports the notion that AC is not simply a localized gallbladder issue but may be a complication of a systemic metabolic disturbance. The finding that diabetes lost its significance in the multivariate analysis implies that its influence is likely mediated primarily through visceral obesity.

This systemic metabolic disturbance involves not only the pro-inflammatory state driven by visceral fat but also complex alterations in neuroendocrine signaling. Gastrointestinal hormones, for instance, are critical regulators of both energy balance and glucose homeostasis. Ghrelin, a key gut hormone primarily produced by the gastric fundus, is notably implicated in this process. Preclinical and clinical studies have demonstrated that ghrelin contributes to poor glycemic control by suppressing insulin secretion and promoting hyperglycemia and insulin resistance (32). This hormonal dysregulation directly exacerbates the state of metabolic syndrome that our study identifies as a high-risk background for AC. Therefore, it is plausible that the systemic pro-inflammatory environment driven by visceral adiposity is compounded by hormonal imbalances, such as those in the ghrelin pathway, which collectively contribute to the pathogenesis of gallstone disease and its progression to acute cholecystitis.

The findings of our study prompt the question of whether targeted interventions aimed at reducing visceral fat and increasing muscle mass could decrease the risk of AC in patients with gallstones?’ The results of this retrospective study provide a strong foundation for future prospective studies and randomized controlled trials. That VFA and SMI are modifiable risk factors opens new avenues for preventive medicine. Asymptomatic gallstone patients with high VFA and low SMI could be identified as high-risk group for developing AC, and more proactive monitoring or lifestyle recommendations (diet, exercise) could be prioritized for these individuals.

Nevertheless, this study has several limitations. Chief among them is its retrospective design, which introduces a potential for patient selection bias. To mitigate this, we included the largest patient cohort available. Furthermore, other metabolic diseases and malignancies, beyond DM, HT, and HL, were not accounted for in the patients’ comorbidities. Additionally, lifestyle factors (such as diet and exercise) were beyond the scope of this study. We could not retrieve data on lifestyle interventions (such as dietary changes) or surgical consultations that may have occurred after the incidental finding of cholelithiasis, which could have influenced the subsequent development of AC.

Although our study was focused on body composition analysis, blood-based inflammatory biomarkers obtainable from blood—such as C-reactive protein, procalcitonin, sedimentation rate, white blood cell count, and IL-6—were excluded due to anticipated data incompleteness. Due to the retrospective nature of the study based on CT reports often focused on other pathologies, specific characteristics of the gallstones (such as size, number, or exact location, e.g., in Hartmann’s pouch) were not systematically available for analysis. These factors are known potential confounders in the progression to AC.

## 5 Conclusion

In conclusion, this study demonstrates that visceral adiposity and sarcopenia are significant, independent predictors for the development of AC in patients with cholelithiasis. Specifically, an increased VFA is a potent risk factor, while a higher skeletal muscle index serves as a protective factor, independent of traditional metrics like BMI. These findings support the hypothesis that AC is not merely a consequence of mechanical obstruction but is, fundamentally linked to a pre-existing pro-inflammatory and metabolic state, driven by an adverse body composition. The identification of this high-risk phenotype—high visceral fat and low muscle mass—offers a new paradigm for risk stratification. Given that these are modifiable risk factors, these results provide a strong rationale for future prospective studies to investigate whether targeted lifestyle or therapeutic interventions can mitigate the risk of this common and serious complication.

## Data Availability

The raw data supporting the conclusions of this article will be made available by the authors, without undue reservation.
